# Dynamic effects of China’s national volume-based procurement on generic drug consistency evaluation: An interrupted time series analysis

**DOI:** 10.1371/journal.pone.0350141

**Published:** 2026-06-10

**Authors:** Xianli Ge, Xiaodong Liu, Weiyu Tian, Jing Peng, Mingyue Zhao, Yu Fang

**Affiliations:** 1 School of Pharmacy, Xi’an Jiaotong University, Xi’an, Shaanxi, China; 2 Center for Drug Safety and Policy Research, Xi’an Jiaotong University, Xi’an, Shaanxi, China‌‌; Islamic Azad University, IRAN, ISLAMIC REPUBLIC OF

## Abstract

**Objectives:**

China’s national volume-based procurement (NVBP) policy uniquely requires the generic drug consistency evaluation (GCE) for generic drugs on the purchase list. It employs large-scale centralized procurement to lower the prices of qualified generic drugs and encourages manufacturers to get GCE certification for their generic drugs. Although it is a fundamental part of the NVBP system, research on the impact of NVBP on manufacturers has only recently emerged and is still not comprehensive enough. This study is the first to systematically evaluate the dynamic effects of the NVBP policy on the quantity of GCE-certified generic drugs and uncover their relationship in terms of both time and quantity.

**Study design:**

Interrupted time series analysis(ITSA).

**Methods:**

The NVBP lists were published by the National Healthcare Security Administration (NHSA). The information of GCE-certified generic drugs was obtained from the databases of the National Medical Products Administration (NMPA) and the Center for Drug Evaluation (CDE). Generic drugs were divided into four categories: all GCE-certified generics; GCE-certified generics of NVBP-listed drugs (NVBP-GCE generics); GCE-certified generics of non-NVBP drugs (non-NVBP GCE generics); and GCE-certified generics from each individual NVBP list.

**Results:**

Overall, the 2018 NVBP intervention resulted in a monthly increase of 2.876 in generics certified by GCE (95% CI: 1.311–4.317). In contrast, the 2016 policy showed no statistical significance. Both NVBP-GCE and non-NVBP GCE generics experienced significant increases, with NVBP-GCE showing a more rapid initial rise. In the analysis of 8 NVBP batches, the 2016 intervention had no significant statistical impact on either the immediate or long – term effects. Conversely, the 2018 intervention significantly increased the quantity of GCE – certified generics in the short term and widened the gap between NVBP – GCE generics and non – NVBP GCE generics. Nevertheless, this effect gradually diminished over time. The quantity of NVBP – GCE generics reached its peak before or at the release of each NVBP list, while non – NVBP GCE generics continued to grow.

**Conclusion:**

The NVBP policy has resulted in a substantial increase in the quantity of GCE – certified generic drugs. From a comprehensive perspective, the growth rates of NVBP-GCE generics and non-NVBP GCE generics were comparable. The maximum number of GCE – certified generic drugs was witnessed either prior to or simultaneously with the release of each round of NVBP procurement documents. After the publication of each NVBP announcement, the number of GCE – certified generics on that particular procurement list started to decrease, whereas the number of non – NVBP GCE generics continued to increase steadily until April 2024.

## 1. Introduction

Generic drugs should be equivalent to innovator drugs in terms of bio – active ingredients, safety, and effectiveness [1]. They are produced by different manufacturers after the patent has expired. These drugs offer advantages in terms of production and cost, reducing healthcare expenses and stabilizing the supply [2]. In China, the annual sales quantity of generic chemical drugs amounts to approximately 900 billion yuan, accounting for roughly 55% of the total drug expenditure [3].

The safety, effectiveness, and bioequivalence of generic drugs are complex to assess [4] and require comparison with the innovator drug through the Generic Consistency Evaluation (GCE) [5]. In China, the National Medical Products Administration (NMPA) conducts GCE. It requires that the 90% confidence interval of key pharmacokinetic parameters should fall within 80.00% − 125.00% for bioequivalence [6].

China’s early generic drug management was lax [4]. Since 2016, GCE certification has been required, yet it has achieved limited success [5]. By 2022, there were 7,974 pharmaceutical companies, and 93,970 approvals were for chemical drugs, 95% of which were generics [3]. Historical and technical factors have led to an excessive number of manufacturers, unstable product quality, high prices, and a large number of unused approvals [3,4,7–9].

China’s National volume-based procurement (NVBP) policy aims to establish a unified, open, and centralized drug procurement market featuring low prices and high quality, with the goal of reducing the expenses of patients and medical insurance funds [10,11]. ‌‌From November 2018 to April 2024, China issued a total of nine NVBP lists, which stipulated that GCE certified generic have equal status with innovator drugs and can share priority procurement rights [12]. A total of 381 drugs were successfully procured [13].

In this study, the metric “quantity of GCE-certified generics” refers to the number of distinct drug products, which takes into account manufacturers, drug composition, dosage, and dosage form combinations. As of April 2024, there are a total of 8,920 GCE-certified generics for 1,206 drugs. Among these, there are 381 NVBP-listed drugs with 6,505 GCE-certified generics and 835 non-NVBP-listed drugs with 2,415 GCE-certified generics.

Existing research on NVBP can be categorized into five thematic areas:

A Drug price reductions resulting from NVBP and their impact on medication accessibility and patient adherence [14–16];
B Efficacy differences between generic drugs and innovator drugs under NVBP [17,18];
C The willingness and extent to which medical institutions and patients adopt GCE-certified generics over innovator drugs [15,19–21];
D Utilization of medical insurance funds for drugs procured centrally [14,22,23];
E The impact of NVBP on manufacturers, particularly concerning patent quantity and the of pricing generic drugs [22,24,25].

While the majority of studies have concentrated on the impacts of NVBP on healthcare systems, medical institutions, patients, and the bioequivalence of generic drugs, research regarding the effect of NVBP on generic drug manufacturers, especially as delineated in Theme E, has only recently emerged.

As a part of the NVBP – manufacturers study, the relationship between the NVBP policy and the GCE – certified generics has not been fully explored. This includes the impact on the timing and quantity of GCE – certified generic drugs, whether it can affect non – NVBP GCE generic drugs, and whether adjustments to the detailed rules of the NVBP policy could influence specific GCE – certified generic drugs. This is the first study to dynamically assess the impacts of multi – round NVBP on GCE certification of generic drugs, which fills an important gap in the previous research.

There are two policy intervention time points in each ITSA study. First, in March 2016, the GCE process was officially implemented for the first time. Second, the time point is when each patch of the NVBP list was introduced.

## 2. Methods

### 2.1. Data sources

Both policy documents and drug marketing authorization records in China—along with the outcomes of the GCE program—are assigned unique, government-issued identification numbers. Policies or drugs lacking official registration on authoritative government platforms are excluded from regulatory recognition and thus deemed ineligible for inclusion. Consequently, these sources provide comprehensive, traceable, and institutionally validated data. For this study, we systematically retrieved historical records using automated web crawlers, supplemented by manual verification against primary source documents; all entries underwent rigorous cross-validation, expert-led manual review, and standardized coding to ensure both accuracy and coverage.

The data on NVBP-listed drugs included in this study were extracted from the official announcements titled *Notice on the Publication of the Results of the National Drug Centralized Procurement Selection,* publicly released by NHSA website between November 2018 to April 2024 [13], a total of 381 drugs were selected across eight procurement rounds. The data on GCE-certified generics—covering the period from January 1, 2013, to April 8, 2024—are classified into two categories, as formally defined in the *Announcement of the NMPA on Relevant Matters Concerning the Quality and Efficacy Consistency Evaluation of Generic Drugs* [26].

A GCE-certified generics refer to legacy generic drugs that had already received marketing authorization prior to the implementation of the GCE program and were subsequently required to undergo separate GCE certification to demonstrate therapeutic equivalence relative to their reference listed drugs. All relevant data were retrieved from the official *GCE Certification Information* database maintained by the Center for Drug Evaluation (CDE) [27].
B Deemed-to-be GCE-certified generics refer to novel generic drugs submitted for marketing authorization on or after July 1, 2020, are required to complete the GCE certification as an integral component of the approval pathway, pursuant to the revised *Regulations on the Administration of Drug Registration*. Consequently, these products are exempt from post-approval GCE certification and are formally classified as “deemed-to-be GCE-certified generics”a designation explicitly established under Articles 8 and 9 of the NMPA’s *Guidelines on Statistical Analysis in Bioequivalence Studies and Technology in Bioequivalence Studies of High- Variation Drugs* [6]. All relevant data were extracted from the official *Delivery Information of Approval Documents for Pharmaceutical Products* database, publicly accessible via the NMPA website [28].

Given that a single GCE certification or marketing authorization can cover multiple dosage forms and strengths of the same active pharmaceutical ingredient from one manufacturer, taking into account the potential variations in clinical use, we calculated and verified each formulation separately. Each of the generic drugs was manually retrieved from certifications and approvals one by one and classified as either Type A or Type B in this study, with no overlapping parts.

Both types hold equivalent legal standing under the NVBP policy and jointly account for a total of 8,920 generic drug products. All data were harmonized using REDCap, with variable definitions—including GCE certification status—standardized across all participating provinces.

### 2.2. Statistical analysis

Interrupted Time Series Analysis (ITSA) [29] is a quasi-experimental design used to assess the causal impact of an intervention on time-series outcomes. The model specification was as follows:


$$\begin{array}{c} Y_{t}=\beta_{\text{0}}+\beta_{\text{1}}T_{\text{1}}+\beta_{\text{2}}X_{t}+\beta_{\text{3}}X_{t}T_{t}+\varepsilon_{t} \end{array}$$

When a study includes both an intervention group and a control group and implements two distinct interventions, the analytical model must be extended to simultaneously account for (1) inter-group comparisons and (2) the time-varying effects of each intervention. The baseline specification of this expanded model is as follows:


$$\begin{array}{c} \begin{array}{c} Y_{it}=\beta_{\text{0}}+\beta_{\text{1}}T_{t}+\beta_{\text{2}}G_{i}+\beta_{\text{3}}\left(T_{t}\times G_{i}\right)+\beta_{\text{4}}I_{1t}+\beta 5\left(I_{1t}\times G_{i}\right)+\beta_{\text{6}}P_{1t}\\ \;\;+\;\beta_{\text{7}}\left(P_{1t}\times G_{i}\right)+\beta_{\text{8}}I_{2t}+\beta_{\text{9}}\left(I_{2t}\times G_{i}\right)+\beta_{10}P_{2t}+\beta_{11}\left(P_{2t}\times G_{i}\right)+\varepsilon_{it} \end{array} \end{array}$$

β_0_:Baseline level (average level before intervention and in the control group)

β_1_T_t_:The linear trend over time before the intervention (control group)

β_2_G_i_:The differences between the intervention group and the control group at the baseline level.

β_3_(T_t_ × G_i_):The difference in the time trend before the intervention between the intervention group and the control group.

β_4_I_1t_:The immediate effect on the control group when the first intervention occurred.

β_5_(I_1t_×G_i_):The additional immediate effect of the first intervention in the intervention group.

β_6_P_1t_:After the first intervention, the trend change of the control group.

β_7_(P_1t_×G_i_):After the first intervention, the additional change in trend of the intervention group compared with the control group.

β_8_I_2t_:The immediate effect on the control group when the second intervention occurred.

β_9_(I_2t_×G_i_):The additional immediate effect of the second intervention in the intervention group.

β_10_P_2t_:After the second intervention, the trend change of the control group.

β_11_(P_2t_×G_i_):After the second intervention, the additional changes in the trend of the intervention group compared with the control group.

ε_it_:Random error term.

Using month as the temporal unit, we assessed the impact of the NVBP policy on GCE-certified generics across three analytical dimensions:

First, the overall effect on the monthly quantity of GCE-certified generics is analyzed, with February 2016 and November 2018 specified as the two intervention time points.

Second, the differential effect between NVBP-listed GCE generics (intervention group) and non-NVBP GCE generics (control group) is analyzed using the same two intervention time points; this comparison isolates the policy’s list-specific influence by leveraging the non-NVBP GCE generics as an internal control.

Third, the cohort – specific impact of each individual NVBP procurement list on GCE – certified generics is analyzed, with February 2016 and the official release date of each respective NVBP list serving as the dual intervention time points. The sixth list is excluded due to its unique design and limited generalizability.

All statistical analyses were carried out using Python (v3.11) in conjunction with the statsmodels and pandas libraries. PyCharm Community Edition 2024.1 was exclusively employed as the integrated development environment (IDE) for coding and debugging.

Using months as the time unit, the impact of the NVBP policy on GCE – certified generics was analyzed from three dimensions. First, the impact on the total quantity of GCE – certified generics was examined with February 2016 and November 2018 as the intervention time points. Second, the difference in impact between NVBP – GCE and non – NVBP GCE generics was investigated. Taking the quantity of non – NVBP GCE generics as the control group and the quantity of NVBP – GCE generics as the experimental group, February 2016 and November 2018 were set as the intervention time points. Third, the impact of each NVBP list on GCE – certified generics was studied, with February 2016 and the release time of each NVBP document as the intervention time points. The sixth list was excluded due to its specificity. Statistical analysis was carried out using PyCharm Community Edition 2024.1.

## 3. Results

### 3.1. Overview

A total of 8,920 GCE – certified generics of 1,206 drugs were included in the study. Among these drugs, 381 drugs purchased by NVBP had 6,505 generics, and 835 drugs not purchased by NVBP had 2,415 generics. Each centrally – purchased drug had an average of 17.07 generics, whereas non – centrally – purchased drugs had an average of 2.89 generics.

**Fig 1:**
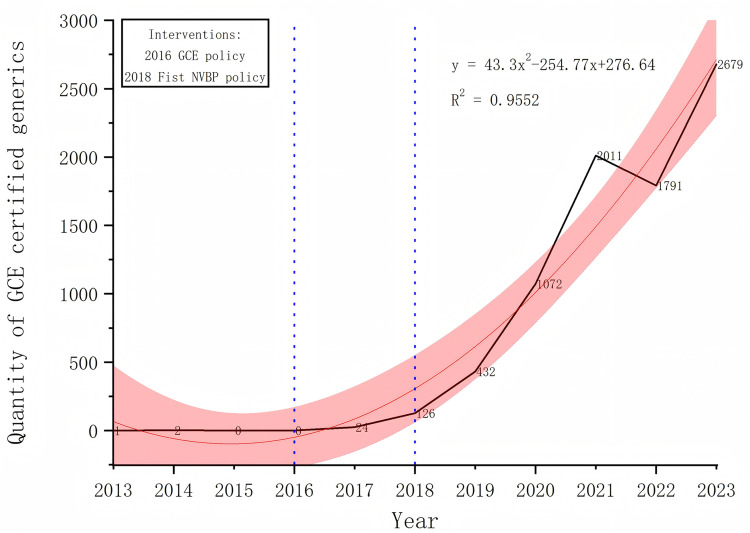
The quantity of GCE certified generics in China from 2013 to 2023.

Using years as the time unit and the annual quantity of GCE – certified generics as the research object, the quantity of all GCE – certified generics from 2012 to 2023 is presented in Fig 1 with a regression polynomial y = 43.3x² - 254.77x + 276.64, where R² = 0.9552. Based on this polynomial, it is estimated that there will be approximately 3380 GCE – certified generics in 2024, around 4170 GCE – certified generics in 2025, and about 5030 GCE – certified generics in 2026. However, due to the limited number of organizations and personnel authorized to evaluate the consistency of generic drugs, it is uncertain whether the annual quantity of GCE – certified generics has reached its peak value.

### 3.2. Interrupted time series analysis(ITSA)

#### 3.2.1. Impact of NVBP policy on the total quantity of GCE certified generics.

**Fig 2:**
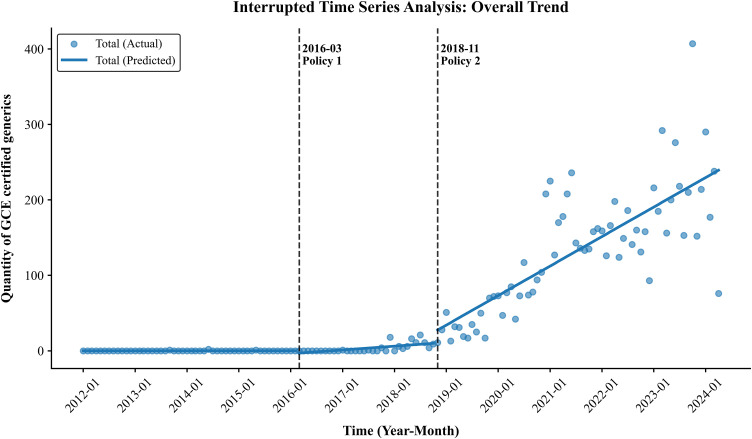
Impact of NVBP policy on the total quantity of GCE certified generics.

Table 1 and Fig 2 illustrate the trends in the quantity of certified GCE generics on a monthly basis before and after the policy intervention.

**Table 1:** Impact of NVBP policy on the total quantity of GCE certified generics.

quantity of GCE certified generics	Coefficient	Std. err.	t	P > |t|	95%CI.lower	95%CI.upper
_t	0.001	0.283	0.000	0.998	−0.555	0.557
_z	0.023	11.450	0.000	0.998	−22.514	22.561
_z_t	−0.005	0.400	−0.010	0.991	−0.791	0.782
_x2016m3	0.200	12.484	0.020	0.987	−24.373	24.772
_x_t2016m3	−0.010	0.619	−0.020	0.987	−1.228	1.208
_z_x2016m3	−3.679	17.655	−0.210	0.835	−38.429	31.072
_z_x_t2016m3	0.487	0.875	0.560	0.578	−1.236	2.209
_x2018m11	−11.589	12.181	−0.950	0.342	−35.566	12.387
_x_t2018m11	1.598	0.577	2.770	0.006	0.462	2.734
_z_x2018m11	40.771	17.227	2.370	0.019	6.863	74.680
_z_x_t2018m11	−0.488	0.816	−0.600	0.550	−2.095	1.118
_cons	0.043	8.096	0.010	0.996	−15.893	15.979

Owing to the absence of group – control restrictions, the emphasis was primarily placed on the significance of the variables and the changes in their time trends. After the first NVBP policy intervention in November 2018, a robust positive effect was demonstrated in the overall time trend (with a 95% confidence interval ranging from 1.311 to 4.317). This outcome suggests that the intervention made a substantial contribution to the improvement of related indicators globally.

In contrast, regarding the main effect of the compulsory GCE certification policy intervention in March 2016 and its time – trend changes, neither achieved statistical significance.

#### 3.2.2. Impact of NVBP policy on the quantity of NVBP-GCE generics and non-NVBP GCE generics.

**Fig 3:**
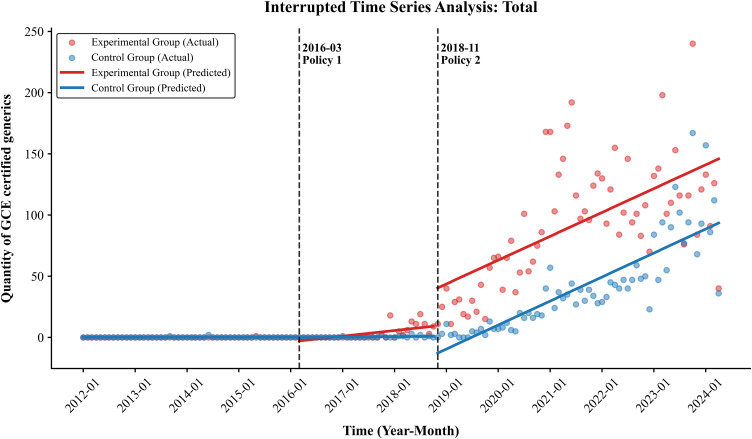
Impact of NVBP policy on the quantity of GCE certified generics and non-NVBP GCE generics.

Overall, Table 2 and Fig 3 indicate that the GCE (2016) intervention had a limited impact on the quantity of GCE generics, while the NVBP (2018) intervention had a significant positive impact on both the time trend and the immediate effect between groups. However, the inter – group difference in the long – term trend was not maintained.

**Table 2:** Impact of NVBP policy on the quantity of GCE certified generics and non-NVBP GCE generics.

quantity of GCE certified generics	Coefficient	Std. err.	t	P > |t|	95%CI.lower	95%CI.upper
_t	0.001	0.283	0.000	0.998	−0.555	0.557
_z	0.023	11.450	0.000	0.998	−22.514	22.561
_z_t	−0.005	0.400	−0.010	0.991	−0.791	0.782
_x2016m3	0.200	12.484	0.020	0.987	−24.373	24.772
_x_t2016m3	−0.010	0.619	−0.020	0.987	−1.228	1.208
_z_x2016m3	−3.679	17.655	−0.210	0.835	−38.429	31.072
_z_x_t2016m3	0.487	0.875	0.560	0.578	−1.236	2.209
_x2018m11	−11.589	12.181	−0.950	0.342	−35.566	12.387
_x_t2018m11	1.598	0.577	2.770	0.006	0.462	2.734
_z_x2018m11	40.771	17.227	2.370	0.019	6.863	74.680
_z_x_t2018m11	−0.488	0.816	−0.600	0.550	−2.095	1.118
_cons	0.043	8.096	0.010	0.996	−15.893	15.979

#### 3.3.3. Impact of each NVBP list on the quantity of GCE certified generics.

(1) The 1st NVBP list (25 drugs)

The first NVBP list encompasses the pilot NVBP in 4 + 7 cities in November 2018 and the expanded NVBP policy in September 2019, with a total of 25 generic drugs. February 2016 was designated as the time of the first policy intervention, and November 2018 as the second. The quantity of non – NVBP GCE generics was selected as the control group, and the quantity of GCE – certified generics of the 1st NVBP listed drugs was chosen as the experimental group.

**Fig 4:**
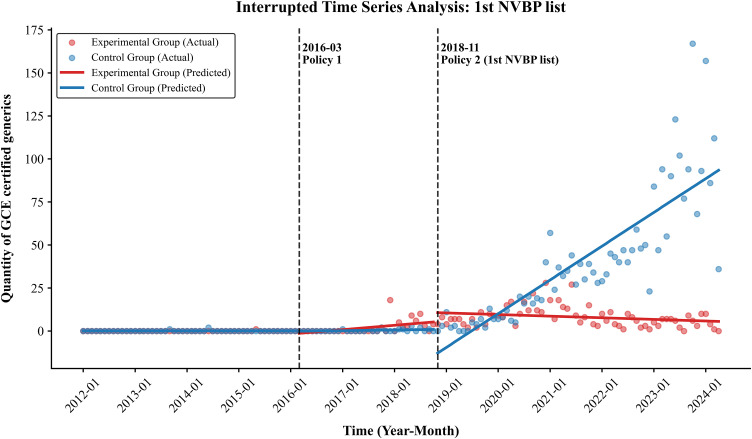
Impact of the 1st NVBP list on the quantity of GCE certified generics.

Table 3 and Fig 4 illustrate the impact of the 1st NVBP list on the quantity of GCE – certified generics.

**Table 3:** Impact of the 1st NVBP list on the quantity of GCE certified generics.

quantity of GCE certified generics	Coefficient	Std. err.	t	P > |t|	95%CI.lower	95%CI.upper
_t	0.000	0.111	0.000	0.997	−0.219	0.220
_z	−0.059	4.485	−0.010	0.989	−8.888	8.769
_z_t	0.001	0.158	0.000	0.997	−0.309	0.311
_x2016m3	−0.109	5.072	−0.020	0.983	−10.093	9.875
_x_t2016m3	0.023	0.244	0.090	0.925	−0.458	0.504
_z_x2016m3	−1.308	7.173	−0.180	0.855	−15.427	12.812
_z_x_t2016m3	0.191	0.346	0.550	0.581	−0.490	0.872
_x2018m11	−13.273	4.931	−2.690	0.008	−22.978	−3.567
_x_t2018m11	1.600	0.229	6.980	0.000	1.149	2.052
_z_x2018m11	18.402	6.973	2.640	0.009	4.676	32.127
_z_x_t2018m11	−1.894	0.324	−5.840	0.000	−2.532	−1.255
_cons	0.049	3.171	0.020	0.988	−6.193	6.292

Overall, the first intervention did not exhibit significant statistical significance in either the immediate or long – term effects on the experimental group. In contrast, the second intervention significantly enhanced the performance of the experimental group in the short term and widened the gap between the two groups. Nevertheless, this effect gradually diminished over time. After the second intervention, the number of GCE – certified generic drugs showed a downward trend.

(2) The 2nd NVBP list (33 drugs)

**Fig 5:**
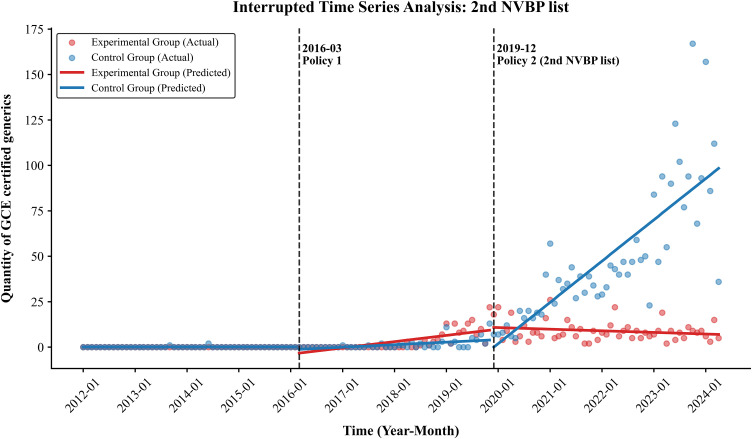
Impact of the 2nd NVBP list on the quantity of GCE certified generics.

February 2016 was taken as the time of the first policy intervention, and December 2019 as the time of the second. The 2nd list includes 33 drugs. Table 4 and Fig 5 show that the results are similar to those of the first list. However, the maximum value of the quantity of GCE – certified generic drugs occurred roughly simultaneously with the second policy intervention.

**Table 4:** Impact of the 2nd NVBP list on the quantity of GCE certified generics.

quantity of GCE certified generics	Coefficient	Std. err.	t	P > |t|	95%CI.lower	95%CI.upper
_t	0.000	0.098	0.000	0.996	−0.192	0.192
_z	−0.050	3.922	−0.010	0.990	−7.770	7.671
_z_t	0.000	0.138	0.000	0.998	−0.272	0.271
_x2016m3	−1.238	4.086	−0.300	0.762	−9.280	6.803
_x_t2016m3	0.115	0.150	0.770	0.444	−0.180	0.411
_z_x2016m3	−2.016	5.778	−0.350	0.727	−13.389	9.356
_z_x_t2016m3	0.171	0.212	0.810	0.421	−0.247	0.589
_x2019m12	−3.991	4.047	−0.990	0.325	−11.958	3.975
_x_t2019m12	1.776	0.145	12.240	0.000	1.490	2.061
_z_x2019m12	5.230	5.724	0.910	0.362	−6.036	16.496
_z_x_t2019m12	−2.136	0.205	−10.410	0.000	−2.540	−1.732
_cons	0.049	2.774	0.020	0.986	−5.410	5.509

(3) The 3rd NVBP list (56 drugs)

**Fig 6:**
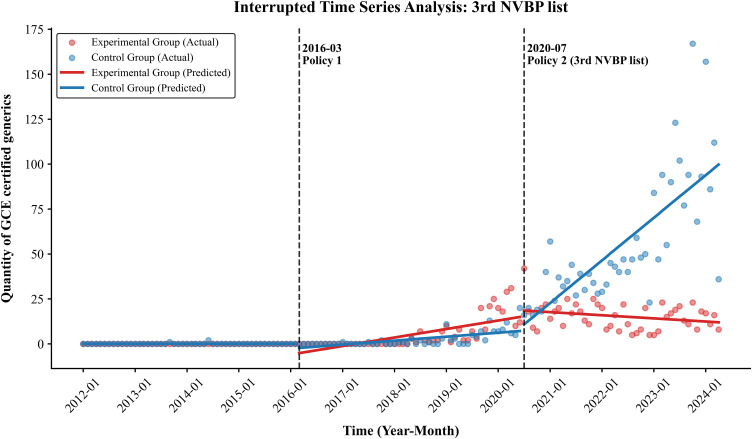
Impact of the 3rd NVBP list on the quantity of GCE certified generics.

Taking July 2020 as the second policy intervention time point, the 3rd list includes 56 drugs. Table 5 and Fig 6 show that the results are similar to those of the second list. However, the maximum value of the quantity of GCE-certified generic drugs occurred exactly simultaneously with the second policy intervention.

**Table 5:** Impact of the 3rd NVBP list on the quantity of GCE certified generics.

quantity of GCE certified generics	Coefficient	Std. err.	t	P > |t|	95%CI.lower	95%CI.upper
_t	0.000	0.103	0.000	0.998	−0.202	0.203
_z	−0.047	4.136	−0.010	0.991	−8.187	8.093
_z_t	−0.001	0.145	0.000	0.997	−0.287	0.286
_x2016m3	−2.322	4.157	−0.560	0.577	−10.505	5.860
_x_t2016m3	0.184	0.141	1.300	0.194	−0.094	0.462
_z_x2016m3	−2.754	5.879	−0.470	0.640	−14.326	8.818
_z_x_t2016m3	0.208	0.200	1.040	0.299	−0.185	0.602
_x2020m7	3.577	4.238	0.840	0.399	−4.764	11.918
_x_t2020m7	1.787	0.152	11.780	0.000	1.488	2.086
_z_x2020m7	−0.271	5.993	−0.050	0.964	−12.067	11.525
_z_x_t2020m7	−2.327	0.214	−10.850	0.000	−2.749	−1.904
_cons	0.051	2.924	0.020	0.986	−5.705	5.807

(4) The 4th NVBP list (45 drugs)

**Fig 7:**
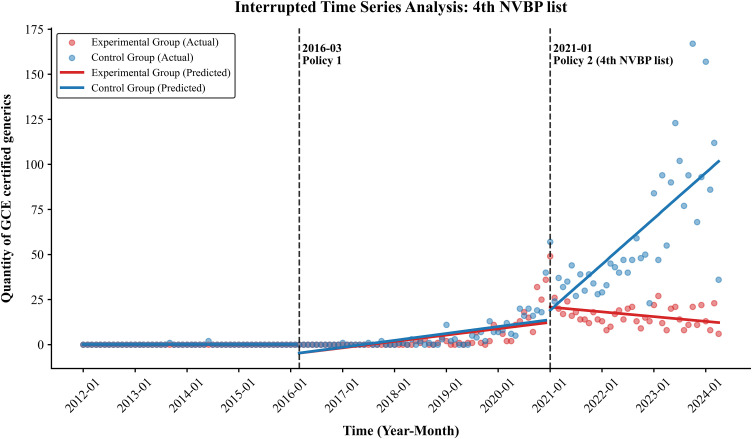
Impact of the 4th NVBP list on the quantity of GCE certified generics.

Taking January 2021 as the second time point for policy intervention, the 4th list comprises 45 drugs. As depicted in Table 6 and Fig 7, the results are similar to those of the 3rd list.

**Table 6:** Impact of the 4th NVBP list on the quantity of GCE certified generics.

quantity of GCE certified generics	Coefficient	Std. err.	t	P > |t|	95%CI.lower	95%CI.upper
_t	−0.001	0.110	−0.010	0.995	−0.217	0.216
_z	−0.049	4.430	−0.010	0.991	−8.770	8.671
_z_t	0.000	0.156	0.000	0.998	−0.307	0.306
_x2016m3	−4.654	4.334	−1.070	0.284	−13.185	3.877
_x_t2016m3	0.317	0.141	2.240	0.026	0.039	0.595
_z_x2016m3	0.019	6.129	0.000	0.998	−12.045	12.083
_z_x_t2016m3	−0.023	0.200	−0.120	0.907	−0.417	0.370
_x2021m1	5.742	4.575	1.260	0.210	−3.263	14.747
_x_t2021m1	1.774	0.178	9.990	0.000	1.425	2.124
_z_x2021m1	2.843	6.470	0.440	0.661	−9.892	15.578
_z_x_t2021m1	−2.291	0.251	−9.130	0.000	−2.786	−1.797
_cons	0.066	3.133	0.020	0.983	−6.100	6.233

(5) 5th NVBP list (63 drugs)

**Fig 8:**
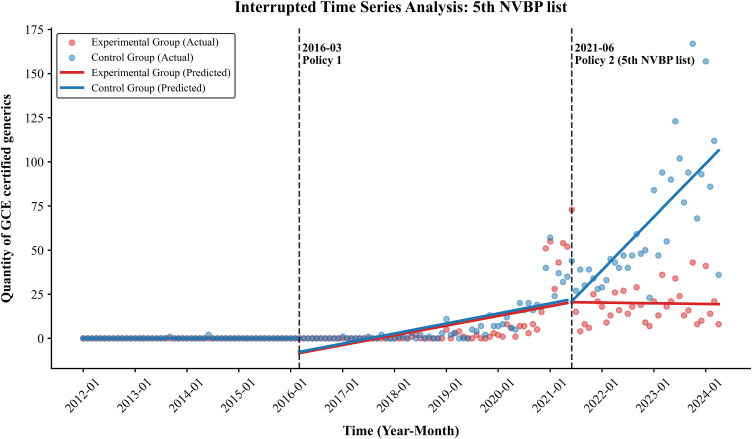
Impact of the 5th NVBP list on the quantity of GCE certified generics.

Taking June 2021 as the time point for policy intervention, the 5th list comprises 63 drugs. As depicted in Table 7 and Fig 8, the results are similar to those of the 3rd list.

**Table 7:** Impact of the 5th NVBP list on the quantity of GCE certified generics.

quantity of GCE certified generics	Coefficient	Std. err.	t	P > |t|	95%CI.lower	95%CI.upper
_t	−0.005	0.142	−0.030	0.972	−0.285	0.275
_z	−0.043	5.735	−0.010	0.994	−11.332	11.246
_z_t	−0.001	0.201	0.000	0.997	−0.396	0.395
_x2016m3	−7.365	5.470	−1.350	0.179	−18.131	3.401
_x_t2016m3	0.472	0.175	2.690	0.008	0.126	0.817
_z_x2016m3	−0.466	7.735	−0.060	0.952	−15.691	14.759
_z_x_t2016m3	−0.021	0.248	−0.080	0.934	−0.509	0.468
_x2021m6	0.296	5.979	0.050	0.961	−11.473	12.065
_x_t2021m6	1.949	0.263	7.410	0.000	1.431	2.466
_z_x2021m6	1.061	8.456	0.130	0.900	−15.583	17.705
_z_x_t2021m6	−2.474	0.372	−6.650	0.000	−3.206	−1.742
_cons	0.134	4.056	0.030	0.974	−7.849	8.117

(6) The 6th NVBP list (16 insulin)

The 6th NVBP was announced in November 2021, including 16 types of insulin.

The 6th NVBP list focuses on insulin procurement, involving a small number of drugs and companies. Therefore, it is not analyzed due to its lack of representativeness.

(7) The 7th NVBP list (61 drugs)

**Fig 9:**
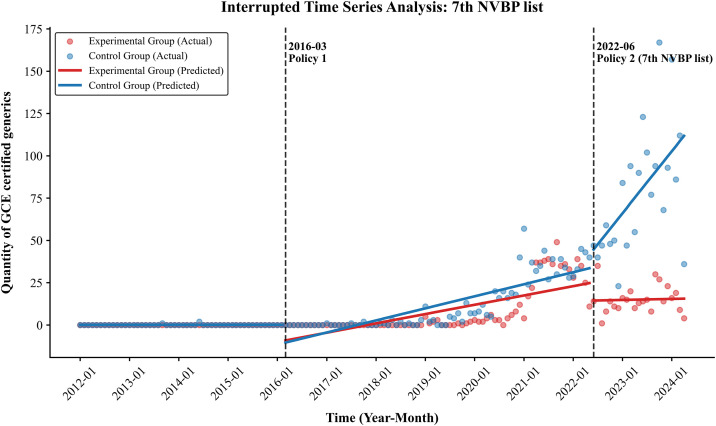
Impact of the 7th NVBP list on the quantity of GCE certified generics.

Taking June 2022 as the second time point for policy intervention, the 7th list comprises 61 drugs. As depicted in Table 8 and Fig 9, the results are similar to those of the 3rd list, except that the maximum value of the generic drugs certified by GCE occurred prior to the second policy intervention.

**Table 8:** Impact of the 7th NVBP list on the quantity of GCE certified generics.

quantity of GCE certified generics	Coefficient	Std. err.	t	P > |t|	95%CI.lower	95%CI.upper
_t	−0.005	0.127	−0.040	0.966	−0.254	0.244
_z	−0.061	5.104	−0.010	0.990	−10.108	9.986
_z_t	0.000	0.179	0.000	0.999	−0.352	0.353
_x2016m3	−10.115	4.720	−2.140	0.033	−19.406	−0.825
_x_t2016m3	0.598	0.145	4.120	0.000	0.312	0.883
_z_x2016m3	1.377	6.675	0.210	0.837	−11.761	14.515
_z_x_t2016m3	−0.140	0.205	−0.680	0.495	−0.543	0.263
_x2022m6	11.560	5.923	1.950	0.052	−0.099	23.219
_x_t2022m6	2.296	0.407	5.640	0.000	1.495	3.096
_z_x2022m6	−21.424	8.376	−2.560	0.011	−37.911	−4.936
_z_x_t2022m6	−2.758	0.575	−4.790	0.000	−3.890	−1.625
_cons	0.140	3.609	0.040	0.969	−6.964	7.245

(8) The eighth NVBP (40 drugs)

**Fig 10:**
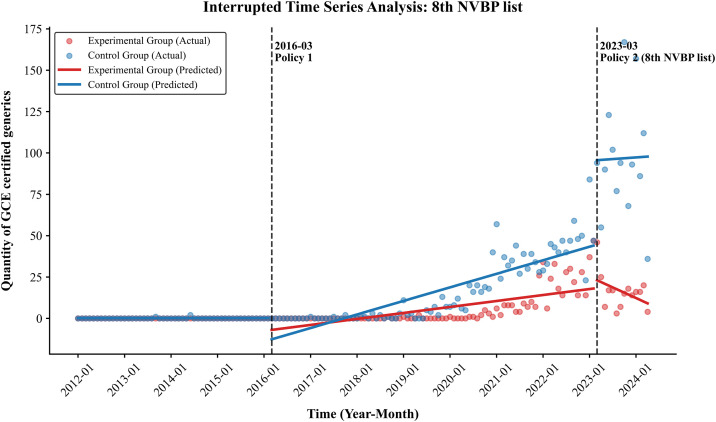
Impact of the 8th NVBP list on the quantity of GCE certified generics.

Taking December 2022 as the time for the second policy intervention, the 8th list comprises 40 drugs. Table 9 and Fig 10 indicate that the results are similar to those of the 3rd list.

**Table 9:** Impact of the 8th NVBP list on the quantity of GCE certified generics.

quantity of GCE certified generics	Coefficient	Std. err.	t	P > |t|	95%CI.lower	95%CI.upper
_t	−0.003	0.112	−0.030	0.976	−0.223	0.216
_z	−0.076	4.495	−0.020	0.986	−8.924	8.771
_z_t	0.001	0.158	0.010	0.994	−0.309	0.312
_x2016m3	−12.528	4.087	−3.070	0.002	−20.573	−4.483
_x_t2016m3	0.685	0.123	5.560	0.000	0.443	0.927
_z_x2016m3	5.666	5.780	0.980	0.328	−5.711	17.043
_z_x_t2016m3	−0.382	0.174	−2.190	0.029	−0.725	−0.039
_x2023m3	51.772	6.194	8.360	0.000	39.581	63.963
_x_t2023m3	−0.725	0.744	−0.980	0.330	−2.189	0.739
_z_x2023m3	−47.113	8.759	−5.380	0.000	−64.354	−29.872
_z_x_t2023m3	−0.661	1.052	−0.630	0.530	−2.731	1.409
_cons	0.109	3.178	0.030	0.973	−6.147	6.365

(9) The 9th NVBP(42 drugs)

Taking October 2023 as the time point for the second policy intervention, the 9th list includes 42 drugs.

**Fig 11:**
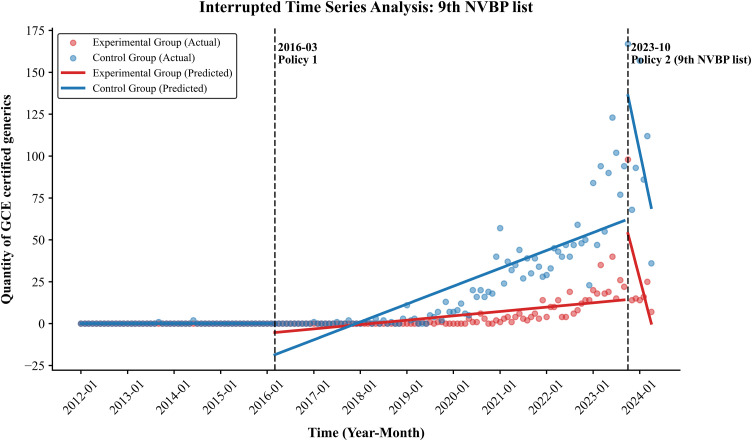
Impact of the 9th NVBP list on the quantity of GCE certified generics.

Table 10 and Fig 11 indicate that at the time of the first intervention, the quantity of GCE – certified generic drugs in the experimental group was significantly lower than that in the control group. This may reflect some challenges in the early – stage implementation of the experimental group. The growth trend of the experimental group increased over time and was significantly better than that of the control group when the second intervention was carried out. After the second intervention, the growth trend of the experimental group showed a significant decline over time, and the maximum value of GCE – certified generic drugs occurred simultaneously with the second policy intervention.

**Table 10:** Impact of the 9th NVBP list on the quantity of GCE certified generics.

quantity of GCE certified generics	Coefficient	Std. err.	t	P > |t|	95%CI.lower	95%CI.upper
_t	−0.026	0.151	−0.170	0.862	−0.323	0.270
_z	−0.357	6.112	−0.060	0.953	−12.388	11.674
_z_t	0.019	0.213	0.090	0.928	−0.400	0.439
_x2016m3	−17.297	5.395	−3.210	0.001	−27.915	−6.678
_x_t2016m3	0.910	0.165	5.530	0.000	0.586	1.234
_z_x2016m3	12.736	7.629	1.670	0.096	−2.281	27.752
_z_x_t2016m3	−0.701	0.233	−3.010	0.003	−1.160	−0.243
_x2023m10	77.854	9.433	8.250	0.000	59.287	96.421
_x_t2023m10	−14.081	2.457	−5.730	0.000	−18.916	−9.246
_z_x2023m10	−28.395	13.340	−2.130	0.034	−54.653	−2.138
_z_x_t2023m10	2.931	3.474	0.840	0.400	−3.907	9.770
_cons	0.468	4.322	0.110	0.914	−8.039	8.975

## 4. Discussion

Globally, generic drugs are a crucial strategy for alleviating the financial burden on both consumers and medical insurance funds while ensuring safe medication use [11]. There exist several policies aimed at controlling the price or quality of generic drugs, such as India’s Jan Aushadhi [30], the FDA’s Generic Drug Competition Action Plan [31] and pan-Canadian Tiered Pricing Framework [32], Moreover, there have been numerous related studies in recent years [33–38].Yet none of these policies have attempted to address both the price and quality of generic drugs simultaneously.

However, China’s NVBP policy uniquely links GCE certification with purchasing rights for NVBP. We thoroughly examined the effects of the 2016 GCE certification policy and the 2018 NVBP policy on the quantity, timing, and trend of GCE-certified generic drugs, as well as whether the policy affects GCE-certified generic drugs not on the NVBP lists.

For all GCE-certified generics, the main effect of the intervention in March 2016 and its time trend have not reached statistical significance. In contrast, a strong positive effect was shown in the overall time trend after the intervention in November 2018.

Regarding the quantity of NVBP – GCE generics and non – NVBP GCE generics, the 2016 intervention had a limited impact on the quantity of GCE generics. In contrast, the 2018 intervention had a significant positive impact on both the time trend and the immediate effect for NVBP – GCE generics and non – NVBP GCE generics. However, the inter – group difference in the long – term trend was not sustained. The quantity of GCE – certified generics on each NVBP list started to decline immediately after the release of each NVBP document. Except for the first NVBP list, the maximum quantity of GCE – certified generics occurred before or at the same time as the release of each NVBP document. That is, the release time of procurement documents is associated with the quantity of GCE – certified generics on the NVBP list, and it is possible to influence the quantity and timing of specific generics to be GCE – certified by adjusting the release time of procurement documents.

The reason for this phenomenon might be that once each batch of NVBP procurement documents is released, it means that the generic drugs with the specific active ingredients can only be purchased from 1 to 3 of those that have already obtained GCE certification. The generic drugs from other manufacturers that are undergoing GCE certification or preparing for it temporarily lose the possibility of winning the bid, thus the quantity of NVBP – GCE generics has dropped significantly. However, the non – NVBP listed generics need to obtain GCE certification to take part in the next batch, so their quantity keeps increasing.

That means, to prevent excessive production of generic drugs from causing resource waste, the government can set up a series of “future procurement pools” based on drug usage, such as for war and disaster, for nearly post – patent innovators, and for infectious diseases. These pools include a list of drugs that are planned for centralized procurement in the next three years. Once the quantity of GCE generic drugs of a specific drug reaches a certain standard, NVBP will commence.

The quantity of GCE-certified generic drugs obtained by different manufacturers varies significantly. This variation makes it challenging for small manufacturers with only a few GCE-certified generic drugs to earn sufficient profits from the NVBP to maintain their operations. The GCE-certified generic drugs of these small manufacturers that fail in the NVBP bid are also unable to compete with the low supply prices of the winning manufacturers for the same products, which may result in actual production halts. The above situation may lead to the de facto monopoly of specific generic drug supplies, thereby increasing the risk of shortages and reducing patient accessibility. Therefore, the trade – off between short – term efficiency and long – term market flexibility needs to be carefully considered. This is the first study to dynamically assess the impacts of multi – round NVBP on GCE certification, filling the gaps in policy iteration research, providing insights for refining the NVBP policy to better manage the supply of GCE – certified generic drugs, and also offering useful implications for LMICs (e.g., African Union’s pooled procurement).

However, this study has certain limitations. First, the quantity of GCE – certified generic drugs fails to fully reflect the actual production capacity of the drug [39–41]. Second, the GCE certifications do not comprehensively represent the real clinical effects [42,43]. Third, the time when the maximum quantity of GCE – certified generic drugs purchased in each NVBP list was observed in this study is merely a phenomenon based on multiple observations and lacks theoretical explanations. Further research and verification are still needed in subsequent studies.

## 5. Conclusion

Generic drugs in China used to lack of standards and regulation, and GCE is regarded as the solution [4,44–46]. The NVBP policy has increased the quantity of generic drugs in China. There was a significant increase in the quantity of all GCE certified generics. The increase rate of NVBP-GCE generics and non-NVBP GCE generics was similar, but the quantity increase of NVBP-GCE generics was greater immediately after the policy intervention. After the release of each NVBP document, the quantity of NVBP GCE generics in that list began to decline, non-NVBP GCE generics keeps increasing till April 2024. The release time of NVBP documents is related to the quantity and timing of GCE certified generics of NVBP list. The maximum value of GCE-certified generic drugs occurred before or simultaneously with the release time of NVBP documents.However, the relationship between the release time of NVBP documents and the quantity and time of generic drugs be certified by GCE needs theoretical explanations and further study.
